# miR-103 Promotes Neurite Outgrowth and Suppresses Cells Apoptosis by Targeting Prostaglandin-Endoperoxide Synthase 2 in Cellular Models of Alzheimer’s Disease

**DOI:** 10.3389/fncel.2018.00091

**Published:** 2018-04-05

**Authors:** Hui Yang, Hongcai Wang, Yongwei Shu, Xuling Li

**Affiliations:** Department of Neurology, The Fourth Affiliated Hospital of Harbin Medical University, Harbin, China

**Keywords:** miR-103, PTGS2, PC12, cerebral cortex neurons, Alzheimer’s disease

## Abstract

miR-103 has been reported to be decreased in brain of transgenic mouse model of Alzheimer’s disease (AD) and in cerebrospinal fluid (CSF) of AD patients, while the detailed mechanism of its effect on AD is obscure, thus this study aimed to investigate the effect of miR-103 expression on neurite outgrowth and cells apoptosis as well as its targets in cellular models of AD. Blank mimic (NC1-mimic), miR-103 mimic, blank inhibitor (NC2-mimic) and miR-103 inhibitor plasmids were transferred into PC12 cellular AD model and Cellular AD model of cerebral cortex neurons which were established by Aβ1–42 insult. Rescue experiment was subsequently performed by transferring Prostaglandin-endoperoxide synthase 2 (PTGS2) and miR-103 mimic plasmid. mRNA and protein expressions were detected by qPCR and Western Blot assays. Total neurite outgrowth was detected by microscope, cells apoptosis was determined by Hoechst/PI assay, and apoptotic markers Caspase 3 and p38 expressions were determined by Western Blot assay. In both PC12 and cerebral cortex neurons cellular AD models, miR-103 mimic increases the total neurite outgrowth compared with NC1-mimic, while miR-103 inhibitor decreases the total neurite outgrowth than NC2-inhibitor. The apoptosis rate was decreased in miR-103 mimic group than NC1-mimic group while increased in miR-103 inhibitor group than NC2-inhibitor group. PTGS2, Adisintegrin and metalloproteinase 10 (ADAM10) and neprilysin (NEP) were selected as target genes of miR-103 by bioinformatics analysis. And PTGS2 was found to be conversely regulated by miR-103 expression while ADAM10 and NEP were not affected. After transfection by PTGS2 and miR-103 mimic plasmid in PC12 cellular AD model, the total neurite growth was shortened compared with miR-103 mimic group, and cells apoptosis was enhanced which indicated PTGS2 mimic attenuated the influence of miR-103 mimic on progression of AD. In conclusion, miR-103 promotes total neurite outgrowth and inhibits cells apoptosis by targeting PTGS2 in cellular models of AD.

## Introduction

Alzheimer’s disease (AD), characterized by neurofibrillary tangles and amyloid β (Aβ) deposits, is one of the most challenging diseases to human health which presents with impaired cognition, memory and language as well as dementia (Chan et al., 2013; Scheltens et al., 2016). Growing evidences disclose that AD affects a great amount of patients worldwide, it’s estimated that 3.12% of total population in United States and 5.05% population in Europe suffer from AD (Beydoun et al., 2015; Niu et al., 2017). As to China, a recent meta-analysis study illuminates that the number of AD patients is quickly increased from 1.93 million in 1990 to 3.71 million in 2000 and to 5.69 million in 2010, which is mainly due to the improved diagnosis of AD, and the prevalence varies from 0.2% to 48.2% in patients aged 55–59 years to patients aged 95–99 years (Chan et al., 2013). Although much progress has been realized in imaging technology, diagnostic biomarkers, treatment drugs, therapy strategy and patients’ care, the prognosis of AD is still far more from satisfaction. Thus, the investigation of pathology of AD and corresponding novel treatment target to prevent, delay or treat AD symptoms is of great need.

Among all the triggers of AD, genetic factor is considered as one of most important elements (Naj et al., 2017). With the advent of genome-wide association studies (GWAS) and next-generation sequencing, more than 30 risk loci related to AD have been identified (Pimenova et al., 2018), which are estimated to explain about 28% of the heritability of liability, 30% of familial risk and over 50% of sibling recurrence risk of developing AD (Cuyvers and Sleegers, 2016). microRNAs (miRNAs), since initially discovered in late time of last decade, have attracted a great amount of attention in neurosciences (Basavaraju and de Lencastre, 2016; Fan et al., 2016). A deal of miRNAs have been proposed to expressed specifically in neurons, where some of them are proved to function in neuronal activities, such as neurite outgrowth and synapse formation (Schratt et al., 2006; Abdelmohsen et al., 2010). In addition, several reports have presented that the genetic ablation of Dicer would lead to neuronal loss, brain shrinkage and inflammation, which is a key enzyme for maturation of miRNA production (Cuellar et al., 2008; Kawase-Koga et al., 2010). A case-control study discloses that 74 up-regulated and 74 down-regulated miRNAs in cerebrospinal fluid (CSF) of AD patients compared to health controls are discovered (Denk et al., 2015). Besides, 20 up-regulated and 32 down-regulated miRNAs are identified in peripheral blood of AD patients by systemic analysis in a recent meta-analysis study (Wu et al., 2016). These indicate miRNAs play crucial functions in AD development and progression.

miR-103, belonging to miR-103/107 family, has been greatly studied in various cancers but not in neurologic diseases (Fasihi et al., 2017; Kfir-Erenfeld et al., 2017; Yang et al., 2017; Zheng et al., 2017). A previous study illuminates that miR-103 improves cancer progress via regulating Wnt signaling pathway in colorectal carcinoma (Fasihi et al., 2017). Another study reveals that miR-103 increases cells proliferation and metastasis through targeting Krüppel-like Factor-4 (KLF4) in gastric cancer (Zheng et al., 2017). While other studies disclose that miR-103 acts as tumor suppressor which inhibit cells proliferation and migration in non-small-cell lung cancer and hematological malignancies (Kfir-Erenfeld et al., 2017; Yang et al., 2017).

Despite of the unclear mechanism of miR-103 function in AD pathology, several studies illustrate that miR-103 is down-regulated in AD patients and models (Yao et al., 2010; Chang et al., 2017; Huynh and Mohan, 2017). A previous animal experiment shows that miR-103 expression is decreased in brain of transgenic mouse model of AD (Yao et al., 2010). Another study applies TaqMan OpenArray Human MicroRNA assay to determine the expression of miRNA profiles in CSF samples, which observes that miR-103 expression is elevated in AD patients compared to health controls (Huynh and Mohan, 2017). Furthermore, a recent secondary systemic analysis presents that miR-103 is estimated to be one of the four most important dysregulated miRNAs in AD patients (Chang et al., 2017). These suggest miR-103 might be involved in the pathology of AD. In addition, miR-107, another key miRNA in miR-103/107 family which shares the same seed region with miR-103, is revealed to be down regulated in AD patients and models as well (Nelson and Wang, 2010). miR-107 regulates AD progress by directly targeting Beta-site amyloid precursor protein cleaving enzyme1 (BACE1), cofilin and even Dicer as previously reported, and it would induce cell cycle arrest in AD model (Wang et al., 2008; Yao et al., 2010; Li et al., 2011; Feng et al., 2012; Jiao et al., 2016). Combining aforementioned reports together, we hypothesized that miR-103 would regulate neuron proliferation and apoptosis in AD models.

However, few study investigating the role of miR-103 in AD etiology has been illuminated, thus we aimed to explore the influence of miR-103 expression on neurite outgrowth and cells apoptosis in cellular AD models, and further validate its target genes in AD.

## Materials and Methods

### Cells Culture

Rat pheochromocytoma cells (PC12 cells) were purchased from Shanghai Institutes for Biological Science (Shanghai, China). After the resuscitation, PC12 cells were cultured in Dulbecco modified Eagle medium (DMEM; Gibco, Gaithersburg, MD, USA) supplemented with 5% fetal bovine serum (FBS; Gibco, Gaithersburg, MD, USA), 10% horse serum (Gibco, Gaithersburg, MD, USA), 100 units/ml penicillin and 100 μg/ml streptomycin. The cells were incubated in a humidified incubator in 95% air and 5% CO_2_ at 37°C.

The brain cortex of SD rat embryos on embryonic day 16 was isolated and subsequently rinsed in Hank’s buffered saline solution, cut into small pieces (0.5–1 mm^3^), digested with trypsin in incubator at 37°C for 15 mins with shaking every 5 mins, dissociated with a fire polished glass pipette and centrifuged to separate un-dissociated tissue. 0.5 ml cell suspensions were then plated in 24-well plates with density of 5 × 10^5^ cells/ml, maintained in serum-free neurobasal medium supplemented with 2% B27 and 0.05 mM glutamine.

The study has been approved by the Animal Ethics Committee of the Fourth Affiliated Hospital of Harbin Medical University, and all related experiments were conducted according to the “Code for the Care and Use of Animals for Scientific Purposes” statement and under the principles of 3R (replacing, refining and reducing).

### PC12 Cells Differentiation by Nerve Growth Factor (NGF)

PC12 cells with density of 1 × 10^5^ cells/ml were plated in 24-well plates and cultured with 20 ng/ml nerve growth factor (NGF; Sigma, USA), 10% FBS (Gibco, Gaithersburg, MD, USA) for 72 h at 37°C with 95% air and 5% CO_2_.

### Preparation of Oligomerized Aβ1–42

Aβ1–42 was purchased from Sigma company (Sigma, USA) and subsequently dissolved in dimethyl sulfoxide (DMSO) at a concentration of 1 mM and stored at −20°C. Prior to the treatment, peptides were pre-incubated at 37°C for 7 days to promote aggregation and then diluted in medium to desired concentrations as described previously. Soluble oligomerized Aβ1–42 peptides (equivalent to 1 mM peptides) were then added to cells to induce damaging effects.

### Aβ1–42 Insult

NGF stimulated PC12 cells and cerebral cortex neurons of SD rat embryos were then treated by 1 mM of soluble oligomerized Aβ1–42 peptides for 24 h to build cellular AD models. And the 3-(4,5-dimethylthiazol-2-yl)-2,5-diphenyl-2H-tetrazolium bromide (MTT) assay was performed to detect the activity of cells viability to validate the establishment of AD models. Cells treated without soluble oligomerized Aβ1–42 peptides were regarded as controls. In addition, miR-103 expression in AD models after Aβ1–42 insult and controls were determined by quantitative polymerase chain reaction (qPCR) assay.

### Plasmids Transfection of miR-103 Mimic and miR-103 Inhibitor

Blank mimic, miR-103 mimic, blank inhibitor and miR-103 inhibitor plasmids were subsequently transferred into PC12 cellular AD model cells and cellular AD model cells of cerebral cortex neurons, and were correspondingly divided into four groups: NC1-mimic, miR-103 mimic, NC2-inhibitor and miR-103 inhibitor groups. After 24 h, Cells neurite outgrowth and apoptosis were detected by microscope, Hoechst/PI, Caspase 3 and p38 expressions for each group. miR-103, prostaglandin-endoperoxide synthase 2 (PTGS2) mRNA, adisintegrin and metalloproteinase 10 (ADAM10) mRNA, neprilysin (NEP) mRNA expressions were determined by qPCR assay, PTGS2, ADAM10 and NEP protein expressions were determined by Western Blot assay in four groups.

PTGS2, ADAM10 and NEP were served as potential targeted genes by these following analysis: (1) target mRNAs of miR-103 were first predicted by miRwalk 2.0 with six or above positive results by 12 methods (miRWalk, Microt4, miRanda, mirbridge, miRDB, miRMap, miRNAMap, Pictar2, PITA, RNA22, RNAhybrid, Targetscan^1^, Dweep et al., 2014); (2) genes correlated with AD pathology or risk were analyzed by DisGenet^2^, Piñero et al., 2017); and (3) potential targeted genes (PTGS2, ADAM10 and NEP) were then analyzed by combining of miR-103 target genes and AD related genes (both existed in miR_103 target genes database and AD related genes database with the top three highest references).

### Plasmids Transfection of miR-103 Mimic and PTGS2 Mimic

In order to determine whether miR-103 regulated AD cells function by targeting PTGS2, first, we determined the PTGS2 expression in PC12 cellular AD model and control by qPCR and Western blot assays; second, the rescue experiment was performed as follows: blank mimic, PTGS2 mimic, miR-103 mimic and PTGS2&miR-103 mimic plasmids were transferred into PC12 cellular AD model cells and divided correspondingly into four groups: NC, PTGS2+, miR-103+, miR-103+ & PTGS2+ groups. After 24 h, Cells neurite outgrowth and apoptosis were detected by microscope, Hoechst/PI, Caspase 3 and p38 expressions for each group. miR-103, PTGS2 mRNA expressions were determined by qPCR assay, PTGS2 protein expression was determined by Western Blot assay.

### MTT Assay

Cells viability was assessed by MTT reagent (Sigma, USA). First, 100 mg of MTT was dissolved in 20 ml PBS to make a 5 mg/ml solution. Next, 10 μl of MTT solution was added to each well, which contained 100 μl culture medium, and incubated at 37°C for 4 h. Then the culture medium in the wells was sucked, and 100 μl of Dimethyl sulfoxide (DMSO) was added to each well, the solution was dissolved for 10 min. Finally, the plates were analyzed using a microplate reader (Molecular Devices, USA) at 490 nm.

### Western Blot Assay

Total proteins were extracted from cells of each group with 1 ml RIPA buffer (Thermo Fisher Scientific, USA). The protein concentration in each sample was then measured using the bicinchoninic acid (BCA) kit (Pierce Biotechnology, Rockford, IL, USA) and compared with the standard curve, the mean of two measurements was calculated for each sample. Twenty microgram protein samples were subjected to sodium dodecyl sulfate polyacrylamide gel electrophoresis (SDS-PAGE) and transferred onto polyvinylidene fluoride membranes (Millipore, Bedford, MA, USA). After blocking with 5% skim milk for 2 h, membranes were incubated with the corresponding primary antibody overnight at 4°C. Then, membranes were incubated with the appropriate secondary antibody for 1 h at room temperature. The bands were visualized using an enhanced chemiluminescence (ECL) kit (Millipore, Bedford, MA, USA) followed by exposure to X-ray film. The antibodies used in this study was summarized in Table 1, and the images of membranes were shown in the Supplementary Data.

**Table 1:** Antibodies used in this study.

Antibody	Company/Country	Dilution ratio
**Primary antibody**
Rabbit polyclonal Anti-Caspase-3 antibody	Abcam/USA	1:2000
Rabbit monoclonal Anti-cleaved Caspase-3 antibody	Abcam/USA	1:2000
Rabbit monoclonal Anti-P38 antibody	Abcam/USA	1:2000
Rabbit polyclonal Anti-P38(phosphoT180+Y182) antibody	Abcam/USA	1:2000
Rabbit monoclonal Anti-GAPDH antibody	Abcam/USA	1:2000
Rabbit polyclonal Anti-neprilysin antibody	Abcam/USA	1:2000	
Rabbit polyclonal to ADAM10	Abcam/USA	1:2000
Rabbit polyclonal to PTGS2	Abcam/USA	1:2000
**Second antibody**
Goat Anti-Rabbit IgG H&L (HRP)	Abcam/USA	1:2000

### qPCR Assay

Expression levels of miRNA and mRNAs were evaluated by qPCR. Total RNA samples from cells was extracted with TRIzol Reagent (Invitrogen, USA), according to the manufacturer’s instructions. Then RNA was quantified by OD 260, and 1 μg of total RNA from each sample was used for cDNA synthesis with transcription kit (TOYOBO, Japan). The cDNA products were subjected to qPCR with SYBR Green kit (KAPA, USA). The PCR amplification was performed as follows: 95°C for 5 min, followed by 40 cycles of 95°C for 5 s, 61°C for 30 s. GAPDH or U6 was used as reference gene. The qPCR results were calculated with the 2^−ΔΔCt^ method as previously described (Arocho et al., 2006). The primers of miRNA and mRNAs were presented in Table 2.

**Table 2:** Primers information.

Gene	Forward primer (5′->3′)	Reverse primer (5′->3′)
GAPDH	CCTTCTCTTGTGACAAAGTGGACA	CATTTGATGTTAGCGGGATCTCG
NEP	GGAAGAAGTGGTTGTTTATGCTCC	AGCATTTCTGGACTCCTTGTAGTT
PTGS2	AACACAGTACACTACATCCTGACC	AACCGTAGTGCACATTGTAAGTTG
ADAM10	GTGCTTGAGAAGAAGAGGAACAAC	TCAGACTTTGACTTGAATGCACAC
U6	CTCGCTTCGGCAGCACA	AACGCTTCACGAATTTGCGT
miR-103	ACACTCCAGCTGGGGGCTTCTTTACAGTGCTG	TGTCGTGGAGTCGGCAATTC

### Morphology Detection and Total Neurite Outgrowth Calculation

Cellular morphology was detected by microscope (Nikon, Japan). Imaging software Presage (Advanced Imaging Concepts, Inc., Brooksville, FL, USA) was used to quantify the total neurite outgrowth per cell which was defined as total length of neurite outgrowth of cells divided by number of cells included.

### Hoechst/PI Assay

Cells death was characterized by double nuclear staining with fluorescent dyes Hoechst 33342 (Sigma, USA) and propidium iodide (PI; Sigma, USA). Briefly, Hoechst 33342 (λex = 350 nm, λem = 461 nm) and PI (λex = 535 nm, λem = 617 nm) were added to the cultured medium at final concentrations of 8 M and 1.5 M, respectively, and cultured at 37°C for 30 min. Images were collected by inversion fluorescence microscope (Nikon, Japan). The total cells and damaged cells were counted under ×200 magnification, and the percentage of damaged cells was calculated. All experiments described in the “Materials and Methods” section were conducted in triplicate.

## Results

### Cells Viability and miR-103 Expression After Aβ1–42 Insult

**Figure 1:**
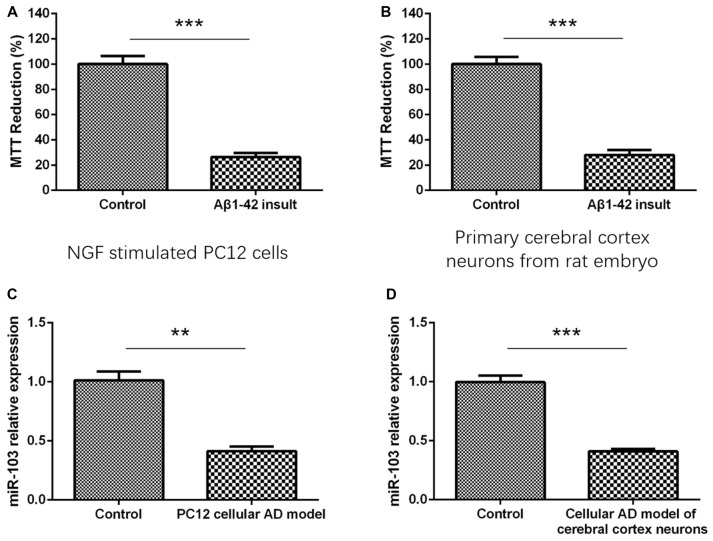
Detection of cells viability by 3-(4,5-dimethylthiazol-2-yl)-2, 5-diphenyl-2H-tetrazolium bromide (MTT) assay after Aβ1–42 insult. **(A)** MTT reduction rate was decreased in Aβ1–42 insult group than control group in nerve growth factor (NGF) stimulated PC12 cells. **(B)** MTT reduction rate was also decreased in Aβ1–42 insult group than control group in primary cerebral cortex neurons from rat embryo. **(C)** miR-103 expression was downregulated in PC12 cellular Alzheimer’s disease (AD) model than control. **(D)** miR-103 expression was reduced in cellular AD model of cerebral cortex neurons compared to control. *P < 0.05, **P < 0.01, ***P < 0.001.

As presented in Figure 1, MTT reduction rate was dramatically decreased in Aβ1–42 insult group compared with control group in both NGF stimulated PC12 cells (Figure 1A) and primary cerebral cortex neurons (Figure 1B), indicating the establishment of cellular AD models. And after construction of AD models, we detected the miR-103 expression between AD models and normal neurons, which illuminated that miR-103 level was decreased in AD models compared to controls in both PC12 cellular AD model (Figure 1C) and cellular AD model of cerebral cortex neurons (Figure 1D).

### miR-103 Expression After Plasmids Transfection

**Figure 2:**
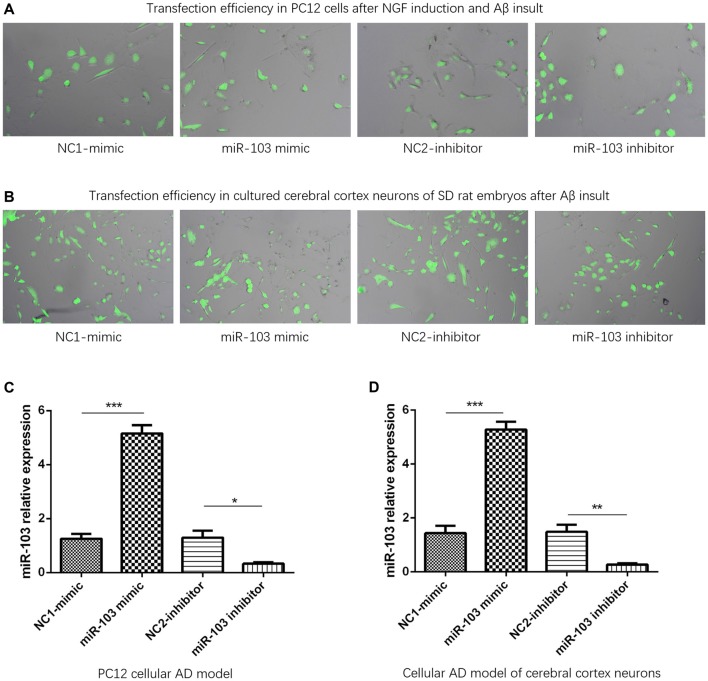
miR-103 expression after transfection. Transfection rates were all above 90% in all four groups in both PC12 cellular AD model **(A)** and cellular AD model of cerebral cortex neurons **(B)** and miR-103 expression was increased in miR-103 mimic group than NC1 while decreased in miR-103 inhibitor group than NC2 **(C,D)**. *P < 0.05, **P < 0.01, ***P < 0.001.

Plasmid transfection efficiency was evaluated by dividing fluorescence positive cells with total cells in 10 fields of microscope through using imageJ software (National Institutes of Health, USA). And we observed that transfection efficiencies were all above 90% in NC1-mimic, miR-103 mimic, NC2-inhibitor and miR-103 inhibitor groups of PC12 cellular AD model (Figure 2A) and cellular AD model of cerebral cortex neurons (Figure 2B). After plasmids transfection, miR-103 expression was increased in miR-103 mimic group compared to NC1-mimic group, and decreased in miR-103 inhibitor group compared with NC2-inhibitor group in both PC12 cellular AD model (Figure 2C) and cellular AD model of cerebral cortex neurons (Figure 2D).

### miR-103 Promoted Neurite Outgrowth

**Figure 3:**
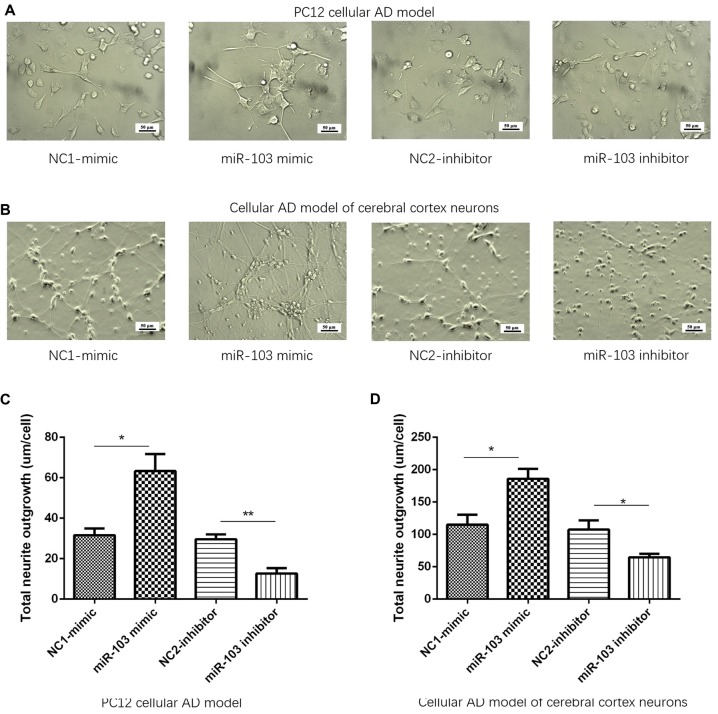
Total neurite outgrowth regulated by miR-103. **(A,B)** Presented cells morphology by microscope at 24 h after transfection: **(A)** PC12 cellular AD model; **(B)** cellular AD model of cerebral cortex neurons. Total neurite outgrowth was increased by miR-103 mimic and decreased by miR-103 inhibitor in both two cellular AD models **(C,D)**. *P < 0.05, **P < 0.01, ***P < 0.001.

After 24 h of plasmids transfection, cellular morphology was determined by microscope in NC1-mimic, miR-103 mimic, NC2-inhibitor and miR-103 inhibitor groups as shown in Figures 3A,B. Subsequently, total neurite outgrowth was calculated. And we found total neurite outgrowth was elevated in miR-103 mimic group compared to NC1-mimic group, while shortened in miR-103 inhibitor group compared with NC2-inhibitor group in both PC12 cellular AD model (Figure 3C) and cellular AD model of cerebral cortex neurons (Figure 3D).

### miR-103 Inhibited Cells Apoptosis

**Figure 4:**
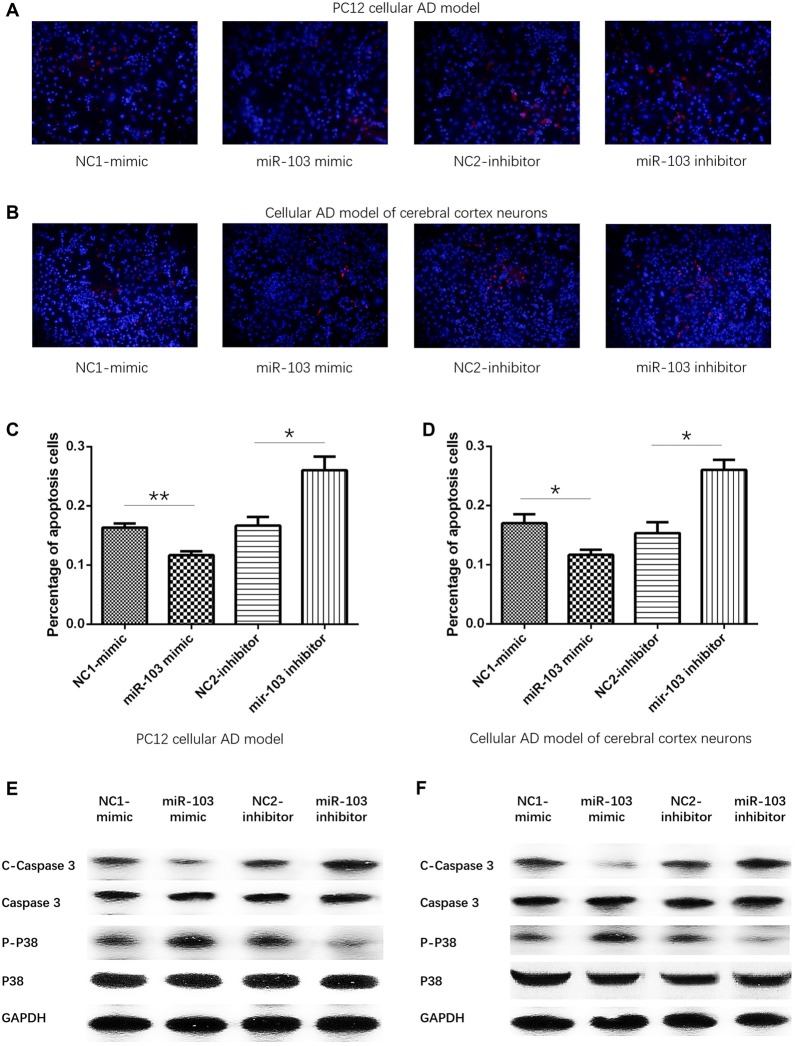
Cells apoptosis regulated by miR-103. Hoechst/PI assay was performed to detect cells apoptosis rate at 24 h after transfection: **(A)** PC12 cellular AD model; **(B)** cellular AD model of cerebral cortex neurons. And miR-103 mimic suppressed apoptosis rate while miR-103 inhibitor improved apoptosis in both two cellular AD models **(C,D)**. In the meanwhile, C-Caspase 3 was decreased in miR-103 mimic group and increased in miR-103 inhibitor group, while P-P38 presented with opposite results **(E,F)**. *P < 0.05, **P < 0.01, ***P < 0.001.

After 24 h of plasmids transfection, Hoechst/PI assay was performed to detect the cells apoptosis as shown in Figures 4A,B. And we observed that percentage of apoptosis cells were decreased in miR-103 mimic group than NC1-mimic group, and enhanced in miR-103 inhibitor group than NC2-inhibitor group in both PC12 cellular AD model (Figure 4C) and cellular AD model of cerebral cortex neurons (Figure 4D). In order to further validate the effect of miR-103 on cells apoptosis in cellular AD models, we subsequently determined the expressions of apoptotic markers including C-Caspase 3 and P-P38. Higher C-Caspase 3 expression stood for higher apoptosis rate while higher P-P38 represented as lower apoptosis rate. And we observed that C-Caspase 3 protein expression was decreased in miR-103 mimic group compared with NC1-mimic group while increased in miR-103 inhibitor group compared to NC2-inhibitor group; P-P38 protein expression was elevated in miR-103 mimic group compared with NC1-mimic group and reduced in miR-103 inhibitor group compared to NC2-inhibitor group in both PC12 cellular AD model (Figure 4E). The results were similar in cellular AD model of cerebral cortex neurons (Figure 4F).

### miR-103 Decreased PTGS2 Expression But Not the Expression of ADAM10 or NEP

**Figure 5:**
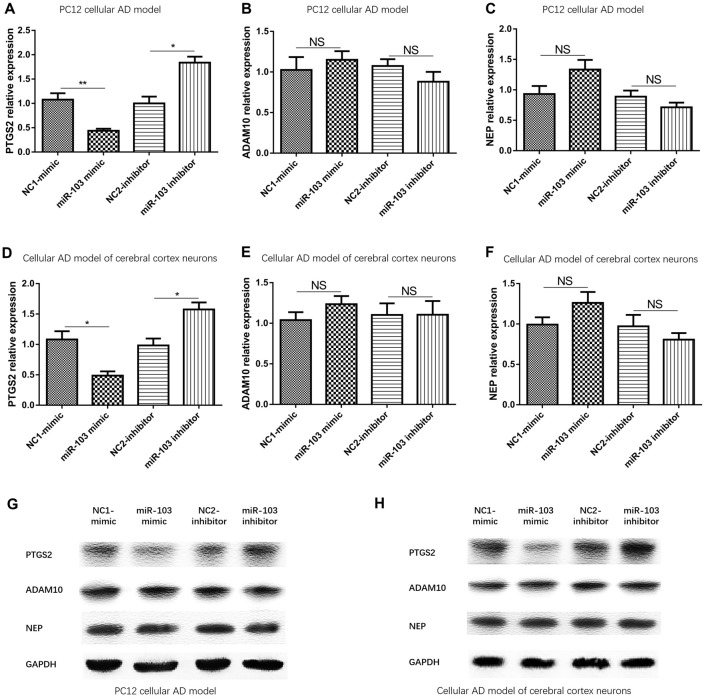
mRNA and protein expression of predicted target genes of miR-103. Prostaglandin-endoperoxide synthase 2 (PTGS2) mRNA and protein levels were both conversely regulated by miR-103 in both PC12 cellular AD model **(A,G)** and cellular AD model of cerebral cortex neurons **(D,H)**. However, Adisintegrin and metalloproteinase 10 (ADAM10) and neprilysin (NEP) expressions were not affected by miR-103 in AD cellular models **(B,C,E,F,G,H)**. *P < 0.05, **P < 0.01, ***P < 0.001.

mRNA and protein expressions of potential targeted genes (PTGS2, ADAM10 and NEP) were subsequently determined by qPCR and Western Blot, which illuminated that PTGS2 mRNA was down regulated in miR-103 mimic group than NC1-mimic group and up regulated in miR-103 inhibitor group than NC2-inhibitor group (Figure 5A), but no difference of ADAM10 (Figure 5B) or NEP (Figure 5C) mRNA was observed in PC12 cellular AD model. In the meanwhile, PTGS2 protein expression was decreased in miR-103 mimic group than NC1-mimic group and increased in miR-103 inhibitor group than NC2-inhibitor group, while ADAM10 and NEP protein expression presented no difference (Figure 5G). The similar results were discovered in cellular AD model of cerebral cortex neurons as well (Figures 5D–F,H).

### miR-103 Inhibited Cells Apoptosis by Targeting PTGS2

**Figure 6:**
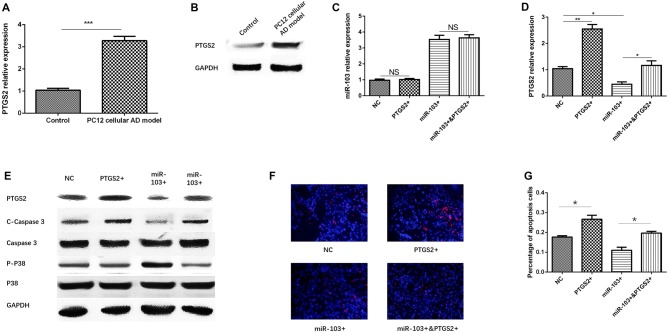
miR-103 suppressed cells apoptosis by targeting PTGS2. PTGS mRNA **(A)** and protein **(B)** expressions were observed to be elevated in PC12 cellular AD model than control. PTGS2 mimic did not affect the expression of miR-103 in PC12 cellular AD model **(C)** while miR-103 mimic conversely regulated PTGS2 expression **(D,E)**. Hoechst/PI assay **(F)** showed PTGS2+ increased cells apoptosis rate compared to NC, and miR-103+&PTGS2+ increased cells apoptosis rate compared with miR-103+ **(G)**. C-Caspase 3 and P-P38 expression also supported these results **(E)**. These indicated PTGS2 attenuated the function of miR-103 on suppressing cells apoptosis in PC12 cellular AD model. *P < 0.05, **P < 0.01, ***P < 0.001.

So as to validate whether miR-103 regulated AD cells function by targeting PTGS2, we first detected PTGS2 mRNA and protein expressions in PC12 cellular AD model and control, which disclosed that both PTGS mRNA (Figure 6A) and protein (Figure 6B) levels were increased in PC12 cellular AD model compared with control. Second, compensating experiment in PC12 cellular AD model was performed, as presented in Figure 6C, no difference of miR-103 expression was found between NC group and PTGS2+ group, or between miR-103+ and miR-103+&PTGS2+ group, indicating PTGS2 mRNA did not regulate miR-103 expression. While both PTGS2 mRNA and protein expressions were elevated in PTGS2+ group than NC group, and in miR-103+&PTGS2+ group than miR-103 group, while reduced in miR-103+ group compared with NC group (Figures 6D,E).

After 24 h of transfection of miR-103 and PTGS2 mimic in PC12 cellular AD model, cells apoptosis was determined by Hoechst/PI assay (Figure 6F), which revealed that percentage of apoptosis was increased in miR-103+&PTGS2+ group compared to miR-103+ group, and in PTGS2+ group compared with NC group (Figure 6G). C-Caspase 3 protein expression was up regulated in miR-103+&PTGS2+ group compared to miR-103+ group, as well as in PTGS2+ group compared with NC group, while the P-P38 presented with the opposite results (Figure 6E). These suggested miR-103 inhibited cells apoptosis by targeting PTGS2.

### miR-103 Increased Neurite Outgrowth by Targeting PTGS2

**Figure 7:**
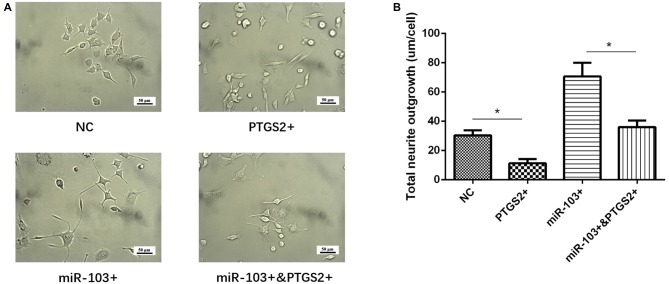
miR-103 improved neurite outgrowth by targeting PTGS2.** (A)** Presented cells morphology in NC, PTGS2+, miR-103+ and miR-103+&PTGS2+ groups, which illuminated that PTGS2 attenuated the function of miR-103 on improving total neurite outgrowth in PC12 cellular AD model **(B)** *P < 0.05, **P < 0.01, ***P < 0.001.

Total neurite outgrowth was calculated at 24 h after transfection of miR-103 and PTGS2 mimic in PC12 cellular AD model (Figure 7A), which disclosed that total neurite outgrowth was decreased in miR-103+&PTGS2+ group than miR-103+ group, as well as in PTGS2+ group than NC group (Figure 7B). These indicated that miR-103 increased neurite outgrowth by targeting PTGS2.

## Discussion

In this present study, we induced cellular AD models based on NGF stimulated PC12 cells and primary cerebral cortex neurons by Aβ1–42 insult, and observed that miR-103 increased total neurite outgrowth and inhibited cells apoptosis in both two cellular AD models, and the function of miR-103 in cellular AD models was independent of ADAM10 or NEP. Furthermore, we performed the compensating experiment of PTGS2 for miR-103 mimic intervention in PC12 cellular AD model, which demonstrated miR-103 improved total neurite outgrowth and suppressed cells apoptosis by targeting PTGS2.

AD, as the most common cause of dementia, is characterized by progressive neuronal loss and inflammation that affect memory, language, behavior and cognition (Scheltens et al., 2016; Winblad et al., 2016). A recent meta-analysis review suggests that approximately 35.6 million people live with dementia in 2010, and the number would increase to 65.7 million in 2030 and 115.4 million in 2050, among which 60% were on account of AD (Prince et al., 2013). In addition to decreased function, memory and cognition, quality of life and so on in AD patients, AD also increases caregiver burden in family members and elevated labor loss as well as social cost (Bertrand et al., 2016; Svendsboe et al., 2016; Deb et al., 2017). Thus AD becomes an increasingly critical issue to human health in the world.

miRNA, as endogenous RNA with about 18–23 nucleotides, regulates protein expression by paring to the mRNA of protein-coding genes and guiding their post-transcriptional repression, which plays critical role in regulating cell differentiation, progression and apoptosis (Thomson and Dinger, 2016). Accumulating evidences disclose that miRNAs involve in the molecular control of development and aging of the brain, while dysregulation of them contributes to the neuropsychiatric disorders including AD (Van den Hove et al., 2014; Basavaraju and de Lencastre, 2016). And several miRNAs including miR-34a/b/c, miR-109, miR-125b, miR-107, miR-181 and so on are illuminated to be involved in the pathology of AD development and progress via targeting multiple genes (Delay et al., 2012; Zhao et al., 2017), and tens of miRNAs have been demonstrated to be biomarker for AD risk and disease monitoring (Hu et al., 2016; Wu et al., 2016). miR-103, located in chromosome 13, has been reported to be decreased in brain of transgenic mouse model of AD as well as in CSF of patients with AD (Yao et al., 2010; Huynh and Mohan, 2017). And deficient expression of miR-103 increases cofilin protein levels in AD model (Yao et al., 2010). In addition, mature miR-103 shares the same seed region with miR-107, the latter is demonstrated to be involved in the pathology of AD by several reports (Nelson and Wang, 2010; Augustin et al., 2012; Jiao et al., 2016). Thus we hypothesized that dysregulated miR-103 would influence the development and progress of AD by regulating disease related genes.

In this present study, we applied two cellular AD models to investigate the effect of miR-103 on total neurite growth and cells apoptosis in AD, which were derived from NGF stimulated PC12 cells and cerebral cortex neurons of SD rat embryos by Aβ1–42 insult as described previously (Fang et al., 2012). Interestingly, we found miR-103 expression was decreased in NGF stimulated PC12 cells and rat embryos neurons after Aβ1–42 insult, which indicated the positive regulating role of Aβ1–42 on miR-103 expression. However, no published report has explored the effect of Aβ1–42 on miR-103, while inversely, miR-103 is observed to directly target ADAM 10, and ADAM 10 is a critical gene in modulating the formation of Aβ1–42 (Jiao et al., 2017). The possible explanation of this result might be that Aβ1–42 promoted neuronal inflammation, which reduced the expression of miR-103 (Ameruoso et al., 2017; Fang et al., 2018; Zhang et al., 2018), nevertheless, this inference needed to be validated in further studies. Subsequently, miR-103 mimic and inhibitor plasmids were used for transfection instead of lentivirus due to the late effect initiation of lentivirus in cells (Shearer and Saunders, 2015). We observed that miR-103 mimic increased total neurite growth and inhibits cells apoptosis in both two cellular AD models, while miR-103 inhibitor realized opposite results. These indicated miR-103 was involved in the pathology of AD by regulating neurite growth and cells apoptosis which was in line with previous clinical studies that disclose miR-103 is down regulated in AD patients and models (Yao et al., 2010; Chang et al., 2017; Huynh and Mohan, 2017). Besides, an animal experiment finds that decreased expression of miR-103 enhances the cofilin protein level in brains of transgenic mouse model of AD (Yao et al., 2010). Another *in vitro* study illuminates that miR-103 regulates p35 which is the main activatory subunit of cyclin-dependent kinase 5 (CDK5) that plays a fundamental role in brain development and functioning, by targeting CKD5R1 (Moncini et al., 2017). Although these two studies do not investigate the direct function of miR-103 in AD cellular models, they provide indirect support for our study that miR-103 influences neurons differentiation and cells apoptosis in AD.

In order to further investigate the mechanism of miR-103 in AD pathology, we next predicted its targeted genes in AD by miRwalk 2.0 database and DisGenet database (Dweep et al., 2014; Piñero et al., 2017). Briefly, we predicted miR-103 target genes by miRwalk and genes related to AD pathology by DisGenet, subsequently combined these two results together, and PTGS2, ADAM10 as well NEP were selected as candidate target genes to be explored. And we found PTGS2 expression was conversely regulated by miR-103 expression in both PC12 cellular AD model and cellular AD model of cerebral cortex neurons, while ADAM 10 and NEP expression was not affected by miR-103. These suggested that miR-103 might function in AD progress by regulating PTGS2 but not ADAM10 or NEP. A previous secondary analysis by computational identification discloses that miR-103 is estimated to target ADAM10 in AD which is in line with our predicted results, however, the regulated effect of miR-103 on ADAM failed to be validated in our experiment in both two cellular AD models (Augustin et al., 2012).

PTGS2, as the gene encoding PTGS2 that is a key enzyme in the conversion of arachidonic acid to prostaglandins involved in inflammation, is demonstrated to play important role in AD development and especially in its progression (Cheng et al., 2013; Toral-Rios et al., 2015). Growing evidences reveal that inflammation may be a third important component which, once initiated in response to neurodegeneration or dysfunction, may actively contribute to AD progression and chronicity (Heneka et al., 2010; Wang et al., 2014). An *in vitro* study discovers that PTGS2-mediated reciprocal regulation of interleukin (IL)-1β and Aβ in glial and neuron cells contributes to the aggravation of AD (Wang et al., 2014). And activation of astrocytes, as a feature of brain inflammation related to AD progress, produces cytokines and PTGS2 which enhances the production of Aβ (Sawikr et al., 2017). In addition, an animal experiment discloses that anti-PTGS2 inhibitor has been observed to improve cognitive decline of AD via concurrently inducing neurogenesis and reducing apoptosis in transgenic mice (Guo et al., 2017). These manifest PTGS2 gene acts as important factor for AD progression. In line with these previous studies, we found PTGS2 mimic decreased total neurite outgrowth and improved cells apoptosis in PC12 cellular AD model, which indicated its role in accelerating AD progression. More importantly, we found PTGS2 mimic attenuated the function of miR-103 mimic on neurite outgrowth and cells apoptosis in PC12 cellular AD model, which suggested miR-103 improved total neurite outgrowth and suppressed cells apoptosis via targeting PTGS2 in AD. This result was in line with previous studies which disclose that miR-103 is decreased in AD patients and models, and the role of PTGS2 on accelerating progression of AD through regulating inflammation (Heneka et al., 2010; Yao et al., 2010; Cheng et al., 2013; Wang et al., 2014; Toral-Rios et al., 2015; Chang et al., 2017; Guo et al., 2017; Huynh and Mohan, 2017; Sawikr et al., 2017).

In conclusion, miR-103 promotes total neurite outgrowth and inhibits cells apoptosis by targeting PTGS2 in cellular models of AD.

## Author Contributions

XL conceived and designed the experiments. HY and HW performed the experiments. HW and YS analyzed the data. HY and YS wrote the main manuscript text. All authors revised the manuscript. All authors reviewed and approved the final manuscript.

## Conflict of Interest Statement

The authors declare that the research was conducted in the absence of any commercial or financial relationships that could be construed as a potential conflict of interest.

## Footnotes

^1^http://zmf.umm.uni-heidelberg.de/apps/zmf/mirwalk2/

^2^www.disgenet.org

## Abbreviations

ADAM10: adisintegrin and metalloproteinase 10
AD: Alzheimer’s disease
Aβ: amyloid β
CDK5: cyclin-dependent kinase 5
CSF: cerebrospinal fluid
DMEM: dulbecco modified eagle medium
DMSO: dimethyl sulfoxide
ECL: enhanced chemiluminescence
FBS: fetal bovine serum
KLF4: Krüppel-like Factor-4
miRNA: microRNA
NEP: neprilysin
NGF: nerve growth factor
qPCR: quantitative polymerase chain reaction
PTGS2: prostaglandin-endoperoxide synthase 2
SDS-PAGE: sodium dodecyl sulfate polyacrylamide gel electrophoresis.

## References

[B1] Abdelmohsen K. Hutchison E. R. Lee E. K. Kuwano Y. Kim M. M. Masuda K.. (2010). miR-375 inhibits differentiation of neurites by lowering HuD levels. Mol. Cell. Biol. 30, 4197–4210. 10.1128/mcb.00316-10 20584986 PMC2937556

[B2] Ameruoso A. Palomba R. Palange A. L. Cervadoro A. Lee A. Di Mascolo D.. (2017). Ameliorating amyloid-β fibrils triggered inflammation via curcumin-loaded polymeric nanoconstructs. Front. Immunol. 8:1411. 10.3389/fimmu.2017.01411 29163489 PMC5671598

[B3] Arocho A. Chen B. Ladanyi M. Pan Q. (2006). Validation of the 2-DeltaDeltaCt calculation as an alternate method of data analysis for quantitative PCR of BCR-ABL P210 transcripts. Diagn. Mol. Pathol. 15, 56–61. 10.1097/00019606-200603000-00009 16531770

[B4] Augustin R. Endres K. Reinhardt S. Kuhn P. H. Lichtenthaler S. F. Hansen J.. (2012). Computational identification and experimental validation of microRNAs binding to the Alzheimer-related gene ADAM10. BMC Med. Genet. 13:35. 10.1186/1471-2350-13-35 22594617 PMC3459808

[B5] Basavaraju M. de Lencastre A. (2016). Alzheimer’s disease: presence and role of microRNAs. Biomol. Concepts 7, 241–252. 10.1515/bmc-2016-0014 27505094 PMC5035151

[B6] Bertrand E. Landeira-Fernandez J. Mograbi D. C. (2016). Metacognition and perspective-taking in Alzheimer’s disease: a mini-review. Front. Psychol. 7:1812. 10.3389/fpsyg.2016.01812 27909421 PMC5112262

[B7] Beydoun M. A. Beydoun H. A. Gamaldo A. A. Rostant O. S. Dore G. A. Zonderman A. B.. (2015). Nationwide inpatient prevalence, predictors and outcomes of Alzheimer’s disease among older adults in the united states, 2002–2012. J. Alzheimers Dis. 48, 361–375. 10.3233/JAD-150228 26402000 PMC4887139

[B8] Chan K. Y. Wang W. Wu J. J. Liu L. Theodoratou E. Car J.. (2013). Epidemiology of Alzheimer’s disease and other forms of dementia in China, 1990–2010: a systematic review and analysis. Lancet 381, 2016–2023. 10.1016/S0140-6736(13)60221-4 23746902

[B9] Chang W. S. Wang Y. H. Zhu X. T. Wu C. J. (2017). Genome-wide profiling of miRNA and mRNA expression in Alzheimer’s disease. Med. Sci. Monit. 23, 2721–2731. 10.12659/msm.905064 28578378 PMC5467707

[B10] Cheng X. R. Cui X. L. Zheng Y. Zhang G. R. Li P. Huang H.. (2013). Nodes and biological processes identified on the basis of network analysis in the brain of the senescence accelerated mice as an Alzheimer’s disease animal model. Front. Aging Neurosci. 5:65. 10.3389/fnagi.2013.00065 24194717 PMC3810591

[B11] Cuellar T. L. Davis T. H. Nelson P. T. Loeb G. B. Harfe B. D. Ullian E.. (2008). Dicer loss in striatal neurons produces behavioral and neuroanatomical phenotypes in the absence of neurodegeneration. Proc. Natl. Acad. Sci. U S A 105, 5614–5619. 10.1073/pnas.0801689105 18385371 PMC2291142

[B12] Cuyvers E. Sleegers K. (2016). Genetic variations underlying Alzheimer’s disease: evidence from genome-wide association studies and beyond. Lancet Neurol. 15, 857–868. 10.1016/s1474-4422(16)00127-7 27302364

[B13] Deb A. Thornton J. D. Sambamoorthi U. Innes K. (2017). Direct and indirect cost of managing Alzheimer’s disease and related dementias in the United States. Expert Rev. Pharmacoecon. Outcomes Res. 17, 189–202. 10.1080/14737167.2017.1313118 28351177 PMC5494694

[B14] Delay C. Mandemakers W. Hébert S. S. (2012). MicroRNAs in Alzheimer’s disease. Neurobiol. Dis. 46, 285–290. 10.1016/j.nbd.2012.01.003 22285895

[B15] Denk J. Boelmans K. Siegismund C. Lassner D. Arlt S. Jahn H. (2015). MicroRNA profiling of CSF reveals potential biomarkers to detect Alzheimer’s disease. PLoS One 10:e0126423. 10.1371/journal.pone.0126423 25992776 PMC4439119

[B16] Dweep H. Gretz N. Sticht C. (2014). miRWalk database for miRNA-target interactions. Methods Mol. Biol. 1182, 289–305. 10.1007/978-1-4939-1062-5_25 25055920

[B17] Fan C. Wu Q. Ye X. Luo H. Yan D. Xiong Y.. (2016). Role of miR-211 in neuronal differentiation and viability: implications to pathogenesis of Alzheimer’s disease. Front. Aging Neurosci. 8:166. 10.3389/fnagi.2016.00166 27458373 PMC4937029

[B19] Fang X. X. Sun G. L. Zhou Y. Qiu Y. H. Peng Y. P. (2018). TGF-β1 protection against Aβ1–42-induced hippocampal neuronal inflammation and apoptosis by TβR-I. Neuroreport 29, 141–146. 10.1097/WNR.0000000000000940 29200096

[B18] Fang M. Wang J. Zhang X. Geng Y. Hu Z. Rudd J. A.. (2012). The miR-124 regulates the expression of BACE1/β-secretase correlated with cell death in Alzheimer’s disease. Toxicol. Lett. 209, 94–105. 10.1016/j.toxlet.2011.11.032 22178568

[B20] Fasihi A. Soltani B. M. Atashi A. Nasiri S. (2017). Introduction of hsa-miR-103a and hsa-miR-1827 and hsa-miR-137 as new regulators of Wnt signaling pathway and their relation to colorectal carcinoma. J. Cell. Biochem. [Epub ahead of print]. 10.1002/jcb.26357 28817181

[B21] Feng L. Xie Y. Zhang H. Wu Y. (2012). miR-107 targets cyclin-dependent kinase 6 expression, induces cell cycle G1 arrest and inhibits invasion in gastric cancer cells. Med. Oncol. 29, 856–863. 10.1007/s12032-011-9823-1 21264532

[B22] Guo J. W. Guan P. P. Ding W. Y. Wang S. L. Huang X. S. Wang Z. Y.. (2017). Erythrocyte membrane-encapsulated celecoxib improves the cognitive decline of Alzheimer’s disease by concurrently inducing neurogenesis and reducing apoptosis in APP/PS1 transgenic mice. Biomaterials 145, 106–127. 10.1016/j.biomaterials.2017.07.023 28865290

[B23] Heneka M. T. O’Banion M. K. Terwel D. Kummer M. P. (2010). Neuroinflammatory processes in Alzheimer’s disease. J. Neural. Transm. (Vienna) 117, 919–947. 10.1007/s00702-010-0438-z 20632195

[B24] Hu Y. B. Li C. B. Song N. Zou Y. Chen S. D. Ren R. J.. (2016). Diagnostic value of microRNA for Alzheimer’s disease: a systematic review and meta-analysis. Front. Aging Neurosci. 8:13. 10.3389/fnagi.2016.00013 26903857 PMC4746262

[B25] Huynh R. A. Mohan C. (2017). Alzheimer’s disease: biomarkers in the genome, blood, and cerebrospinal fluid. Front. Neurol. 8:102. 10.3389/fneur.2017.00102 28373857 PMC5357660

[B26] Jiao T. Yao Y. Zhang B. Hao D. C. Sun Q. F. Li J. B.. (2017). Role of MicroRNA-103a targeting ADAM10 in abdominal aortic aneurysm. Biomed Res. Int. 2017:9645874. 10.1155/2017/9645874 28357407 PMC5357520

[B27] Jiao Y. Kong L. Yao Y. Li S. Tao Z. Yan Y.. (2016). Osthole decreases β amyloid levels through up-regulation of miR-107 in Alzheimer’s disease. Neuropharmacology 108, 332–344. 10.1016/j.neuropharm.2016.04.046 27143098

[B28] Kawase-Koga Y. Low R. Otaegi G. Pollock A. Deng H. Eisenhaber F.. (2010). RNAase-III enzyme Dicer maintains signaling pathways for differentiation and survival in mouse cortical neural stem cells. J. Cell Sci. 123, 586–594. 10.1242/jcs.059659 20103535 PMC2818196

[B29] Kfir-Erenfeld S. Haggiag N. Biton M. Stepensky P. Assayag-Asherie N. Yefenof E. (2017). miR-103 inhibits proliferation and sensitizes hemopoietic tumor cells for glucocorticoid-induced apoptosis. Oncotarget 8, 472–489. 10.18632/oncotarget.13447 27888798 PMC5352135

[B30] Li X. Zhang Y. Shi Y. Dong G. Liang J. Han Y.. (2011). MicroRNA-107, an oncogene microRNA that regulates tumour invasion and metastasis by targeting DICER1 in gastric cancer. J. Cell. Mol. Med. 15, 1887–1895. 10.1111/j.1582-4934.2010.01194.x 21029372 PMC3918045

[B31] Moncini S. Lunghi M. Valmadre A. Grasso M. Del Vescovo V. Riva P.. (2017). The miR-15/107 family of microRNA genes regulates CDK5R1/p35 with implications for Alzheimer’s disease pathogenesis. Mol. Neurobiol. 54, 4329–4342. 10.1007/s12035-016-0002-4 27343180

[B32] Naj A. C. Schellenberg G. D. Alzheimer’s Disease Genetics Consortium (ADGC). (2017). Genomic variants, genes, and pathways of Alzheimer’s disease: an overview. Am. J. Med. Genet. B Neuropsychiatr. Genet. 174, 5–26. 10.1002/ajmg.b.32499 27943641 PMC6179157

[B33] Nelson P. T. Wang W. X. (2010). MiR-107 is reduced in Alzheimer’s disease brain neocortex: validation study. J. Alzheimers Dis. 21, 75–79. 10.3233/JAD-2010-091603 20413881 PMC2910235

[B34] Niu H. Álvarez-Álvarez I. Guillén-Grima F. Aguinaga-Ontoso I. (2017). Prevalence and incidence of Alzheimer’s disease in Europe: a meta-analysis. Neurologia 32, 523–532. 10.1016/j.nrl.2016.02.016 27130306

[B35] Pimenova A. A. Raj T. Goate A. M. (2018). Untangling genetic risk for Alzheimer’s disease. Biol. Psychiatry 83, 300–310. 10.1016/j.biopsych.2017.05.014 28666525 PMC5699970

[B36] Piñero J. Bravo A. Queralt-Rosinach N. Gutiérrez-Sacristán A. Deu-Pons J. Centeno E.. (2017). DisGeNET: a comprehensive platform integrating information on human disease-associated genes and variants. Nucleic Acids Res. 45, D833–D839. 10.1093/nar/gkw943 27924018 PMC5210640

[B37] Prince M. Bryce R. Albanese E. Wimo A. Ribeiro W. Ferri C. P. (2013). The global prevalence of dementia: a systematic review and metaanalysis. Alzheimers Dement. 9, 63.e2–75.e2. 10.1016/j.jalz.2012.11.007 23305823

[B38] Sawikr Y. Yarla N. S. Peluso I. Kamal M. A. Aliev G. Bishayee A. (2017). Neuroinflammation in Alzheimer’s disease: the preventive and therapeutic potential of polyphenolic nutraceuticals. Adv. Protein Chem. Struct. Biol. 108, 33–57. 10.1016/bs.apcsb.2017.02.001 28427563

[B39] Scheltens P. Blennow K. Breteler M. M. de Strooper B. Frisoni G. B. Salloway S.. (2016). Alzheimer’s disease. Lancet 388, 505–517. 10.1016/S0140-6736(15)01124-1 26921134

[B40] Schratt G. M. Tuebing F. Nigh E. A. Kane C. G. Sabatini M. E. Kiebler M.. (2006). A brain-specific microRNA regulates dendritic spine development. Nature 439, 283–289. 10.1038/nature04367 16421561

[B41] Shearer R. F. Saunders D. N. (2015). Experimental design for stable genetic manipulation in mammalian cell lines: lentivirus and alternatives. Genes Cells 20, 1–10. 10.1111/gtc.12183 25307957

[B42] Svendsboe E. Terum T. Testad I. Aarsland D. Ulstein I. Corbett A.. (2016). Caregiver burden in family carers of people with dementia with Lewy bodies and Alzheimer’s disease. Int. J. Geriatr. Psychiatry 31, 1075–1083. 10.1002/gps.4433 26765199

[B43] Thomson D. W. Dinger M. E. (2016). Endogenous microRNA sponges: evidence and controversy. Nat. Rev. Genet. 17, 272–283. 10.1038/nrg.2016.20 27040487

[B44] Toral-Rios D. Franco-Bocanegra D. Rosas-Carrasco O. Mena-Barranco F. Carvajal-Garcia R. Meraz-Rios M. A.. (2015). Evaluation of inflammation-related genes polymorphisms in Mexican with Alzheimer’s disease: a pilot study. Front. Cell. Neurosci. 9:148. 10.3389/fncel.2015.00148 26041990 PMC4435067

[B45] Van den Hove D. L. Kompotis K. Lardenoije R. Kenis G. Mill J. Steinbusch H. W.. (2014). Epigenetically regulated microRNAs in Alzheimer’s disease. Neurobiol. Aging 35, 731–745. 10.1016/j.neurobiolaging.2013.10.082 24238656

[B46] Wang P. Guan P. P. Wang T. Yu X. Guo J. J. Wang Z. Y. (2014). Aggravation of Alzheimer’s disease due to the COX-2-mediated reciprocal regulation of IL-1β and Aβ between glial and neuron cells. Aging Cell 13, 605–615. 10.1111/acel.12209 24621265 PMC4326948

[B47] Wang W. X. Rajeev B. W. Stromberg A. J. Ren N. Tang G. Huang Q.. (2008). The expression of microRNA miR-107 decreases early in Alzheimer’s disease and may accelerate disease progression through regulation of β-site amyloid precursor protein-cleaving enzyme 1. J. Neurosci. 28, 1213–1223. 10.1523/JNEUROSCI.5065-07.2008 18234899 PMC2837363

[B48] Winblad B. Amouyel P. Andrieu S. Ballard C. Brayne C. Brodaty H.. (2016). Defeating Alzheimer’s disease and other dementias: a priority for European science and society. Lancet Neurol. 15, 455–532. 10.1016/S1474-4422(16)00062-4 26987701

[B49] Wu H. Z. Ong K. L. Seeher K. Armstrong N. J. Thalamuthu A. Brodaty H.. (2016). Circulating microRNAs as biomarkers of Alzheimer’s disease: a systematic review. J. Alzheimers Dis. 49, 755–766. 10.3233/JAD-150619 26484928

[B50] Yang D. Wang J. J. Li J. S. Xu Q. Y. (2017). MiR-103 functions as a tumor suppressor by directly targeting programmed cell death 10 in NSCLC. Oncol. Res. [Epub ahead of print]. 10.3727/096504017x15000757094686 28734041 PMC7844823

[B51] Yao J. Hennessey T. Flynt A. Lai E. Beal M. F. Lin M. T. (2010). MicroRNA-related cofilin abnormality in Alzheimer’s disease. PLoS One 5:e15546. 10.1371/journal.pone.0015546 21179570 PMC3002958

[B52] Zhang Z. Wu S. Muhammad S. Ren Q. Sun C. (2018). miR-103/107 promote ER stress mediated apoptosis via targeting the wnt3a/β-catenin /ATF6 pathway in preadipocytes. J. Lipid Res. [Epub ahead of print]. 10.1194/jlr.m082602 29483204 PMC5928437

[B53] Zhao J. Yue D. Zhou Y. Jia L. Wang H. Guo M.. (2017). The role of MicroRNAs in Aβ deposition and tau phosphorylation in Alzheimer’s disease. Front. Neurol. 8:342. 10.3389/fneur.2017.00342 28769871 PMC5513952

[B54] Zheng J. Liu Y. Qiao Y. Zhang L. Lu S. (2017). miR-103 promotes proliferation and metastasis by targeting KLF4 in gastric cancer. Int. J. Mol. Sci. 18:E910. 10.3390/ijms18050910 28445396 PMC5454823

