# Supplementary material for: miR-103 Promotes Neurite Outgrowth and Suppresses Cells Apoptosis by Targeting Prostaglandin-Endoperoxide Synthase 2 in Cellular Models of Alzheimer’s Disease
Source: Front Cell Neurosci. 2018 Apr 5;12:91. doi: 10.3389/fncel.2018.00091 (PMC5895658; doi:10.3389/fncel.2018.00091)
Supplement: Supplementary file 1 [file Data_Sheet_1.DOCX]

***Supplementary Material***

# MiR-103 promotes neurite outgrowth and suppresses cells apoptosis by targeting prostaglandin-endoperoxide synthase 2 in cellular models of Alzheimer's disease

**Hui Yang, Hongcai Wang, Yongwei Shu, Xuling Li^*^**

***Correspondence:** Xuling Li: [lixulling@163.com](mailto:lixulling@163.com)

# Supplementary Data


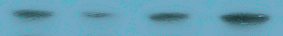


Fig 4E-1


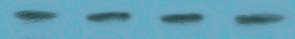


Fig 4E-2


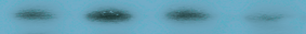


Fig 4E-3


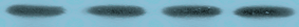


Fig 4E-4


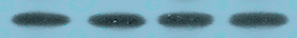


Fig 4E-5


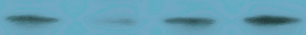


Fig 4F-1


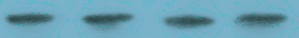


Fig 4F-2


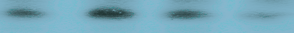


Fig 4F-3


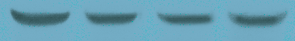


Fig 4F-4


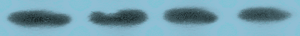


Fig 4F-5


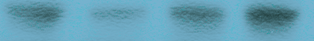


Fig 5G-1


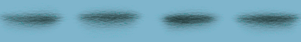


Fig 5G-2


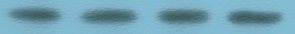


Fig 5G-3


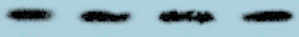


Fig 5G-4


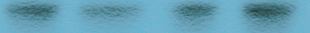


Fig 5H-1


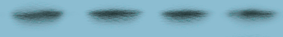


Fig 5H-2


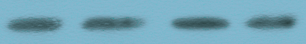


Fig 5H-3


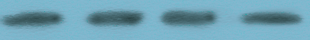


Fig 5H-4


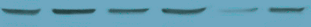


Fig 6B-1


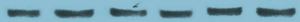


Fig 6B-2


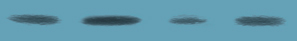


Fig 6E-1


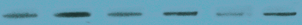


Fig 6E-2


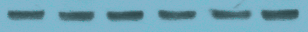


Fig 6E-3


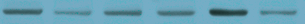


Fig 6E-4


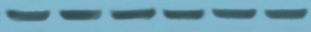


Fig 6E-5


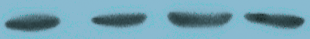


Fig 6E-6
